# Microglial Sirtuin 2 Shapes Long-Term Potentiation in Hippocampal Slices

**DOI:** 10.3389/fnins.2020.00614

**Published:** 2020-06-18

**Authors:** Joana Sa de Almeida, Mariana Vargas, João Fonseca-Gomes, Sara Ramalho Tanqueiro, Rita F. Belo, Catarina Miranda-Lourenço, Ana M. Sebastião, Maria José Diógenes, Teresa F. Pais

**Affiliations:** ^1^Division of Development and Growth, Department of Woman, Child and Adolescent, University Hospitals of Geneva, Geneva, Switzerland; ^2^Instituto de Farmacologia e Neurociências, Faculdade de Medicina, Universidade de Lisboa, Lisbon, Portugal; ^3^Instituto de Medicina Molecular João Lobo Antunes, Faculdade de Medicina, Universidade de Lisboa, Lisbon, Portugal; ^4^Instituto Gulbenkian de Ciência, Oeiras, Portugal

**Keywords:** sirtuin 2, long-term potentiation, microglia, neuroinfalmmation, memantine

## Abstract

Microglial cells have emerged as crucial players in synaptic plasticity during development and adulthood, and also in neurodegenerative and neuroinflammatory conditions. Here we found that decreased levels of Sirtuin 2 (Sirt2) deacetylase in microglia affects hippocampal synaptic plasticity under inflammatory conditions. The results show that long-term potentiation (LTP) magnitude recorded from hippocampal slices of wild type mice does not differ between those exposed to lipopolysaccharide (LPS), a pro-inflammatory stimulus, or BSA. However, LTP recorded from hippocampal slices of microglial-specific Sirt2 deficient (Sirt2^–^) mice was significantly impaired by LPS. Importantly, LTP values were restored by memantine, an antagonist of N-methyl-D-aspartate (NMDA) receptors. These results indicate that microglial Sirt2 prevents NMDA-mediated excitotoxicity in hippocampal slices in response to an inflammatory signal such as LPS. Overall, our data suggest a key-protective role for microglial Sirt2 in mnesic deficits associated with neuroinflammation.

## Introduction

Neuroinflammation generally refers to the noxious effects caused by immunological activation of microglia and astrocytes in various diseases of the central nervous system (CNS) and also in aging (Streit et al., 2004).

Microglia are primary innate immune cells, the resident macrophages of the CNS that constantly survey the microenvironment. They are known to play an important role in regulating brain development and in synaptic plasticity, controlling synapse formation, function and elimination during development and adulthood (Lenz and Nelson, 2018; Lee and Chung, 2019). Additionally, abnormal microglial activation, through the activation of the transcription factor NF-κB (Glass et al., 2010), a master regulator of the inflammatory and neurotoxic gene response (Streit et al., 2004; Glass et al., 2010), contributes to the initiation and progression of various neurodegenerative diseases (Glass et al., 2010). Once the delicate neuro-glial interactive balance is compromised, CNS synaptic transmission, plasticity and memory are also affected (Streit et al., 2004; Bains and Oliet, 2007; Di Filippo et al., 2008). Indeed, the release of pro-inflammatory molecules such as IL-1β may result in synaptic dysfunction. Accordingly, there are evidences that both peripheral and central inflammation are associated with impairments of synaptic plasticity in particular of hippocampal CA1 long-term potentiation (LTP) which is dependent on glutamate NMDA receptors activation (Di Filippo et al., 2013). LTP is considered an electro-physiological model for the basic mechanisms involved in learning and memory formation (Bliss and Collingridge, 1993; Martin and Shapiro, 2000) known to be impaired in models of neurodegenerative diseases characterized by mnesic deficits (Martin and Shapiro, 2000; Lynch, 2004).

Sirtuins are a seven-member (SIRT1-SIRT7) family of NAD^+^-dependent lysine deacetylases in humans and other mammals that can act on different substrates and regulate a variety of cellular functions, comprising genome maintenance, longevity and metabolism (Westphal et al., 2007; Yamamoto et al., 2007; Vassilopoulos et al., 2011). They have been shown to hold protective effects against age-related diseases such as: cancer, diabetes, cardiovascular and neurodegenerative diseases, including Alzheimer’s disease (AD) and Parkinson’s disease (PD) (Donmez and Outeiro, 2013). Sirtuins interfere with multiple key processes of neurodegeneration, namely by preventing the accumulation of toxic protein aggregates and restoring protein homeostasis (Donmez and Outeiro, 2013), by increasing transcription of important genes for learning and memory with an impact in neural plasticity (Herskovits and Guarente, 2014), by reducing oxidative stress through activation of mitochondrial functions (Osborne et al., 2016) and by suppressing sustained chronic inflammation via inhibition of NF-κB and through epigenetic modifications (Han, 2009; Min et al., 2013).

Sirt2 is the most abundant sirtuin in the brain (Maxwell et al., 2011). It is primarily a cytoplasmic protein that can transiently shuttle into the nucleus during mitosis but little is known about its precise function (Harting and Knoll, 2010; de Oliveira et al., 2012). An increasing number of studies have reported that Sirt2 is involved in the regulation of inflammatory diseases (Yu et al., 2016; Yuan et al., 2016). In particular, Sirt2 has been shown to be a potential regulator of brain inflammatory responses mediated by microglia, holding a key inhibitory role in microglia−mediated inflammation and neurotoxicity (Pais et al., 2013). Upon pro-inflammatory stimuli, to counteract microglial overactivation, Sirt2 is dephosphorylated, which enhances its deacetylase activity. Sirt2 deacetylates NF-κB (which is found hyperacetylated in the absence of Sirt2), inhibiting thus NF-κB-dependent pro-inflammatory cytokine expression and preventing, this way, excessive transcription of genes that are related to aging and inflammation (Pais et al., 2013; Lo Sasso et al., 2014; Rothgiesser et al., 2019). Therefore, Sirt2 might function as a “gatekeeper,” preventing excessive microglial activation and restraining its deleterious effects through NF-κB deacetylation. Indeed, it has been shown that intra-cortical injection of LPS decreases Sirt2 levels in the brain of wild type mice and is associated with increased nitrotyrosination, neurotoxicity and neuronal cell death in Sirt2 deficient mice (Fernandes et al., 2012; Kim et al., 2013; Pais et al., 2013).

Additionally, Sirt2 was shown to alleviate neuropathic pain (Zhang and Chi, 2018) and traumatic brain injury (Yuan et al., 2016) associated with inhibition of NF-κB signaling and neuroinflammation. On the other hand, deletion of Sirt2 resulted in depressive-like behaviors and impaired hippocampal neurogenesis (Liu et al., 2015).

Given that Sirt2 plays a key role in microglia-mediated inflammation, and that LTP can be affected by neuroinflammation, we aimed to investigate the impact of microglial-Sirt2 deficiency on hippocampal LTP. Our findings revealed that, upon LPS inflammatory stimulus, Sirt2-deficiency in microglia leads to an LTP impairment in hippocampal slices, which can be reversed by prior exposition of hippocampal slices to memantine, an antagonist of NMDA receptor. Our results suggest that microglial Sirt2 prevents NMDA-mediated excitotoxicity in hippocampal slices stimulated with LPS, revealing thus a key-protective role for microglial Sirt2 in mnesic deficits associated with neuroinflammation.

## Materials and Methods

### Animals

Myeloid-specific Sirt2 deficient mice, LysM^Cre/wt^Sirt2^flox/flox^, were generated at Instituto de Medicina Molecular João Lobo Antunes (IMM) by crossing LysM^Cre/wt^ mice (The Jackson Laboratory) with Sirt2^flox/flox^ mice (Dr. Auwerx, Laboratory of Integrative and Systems Physiology, Ecole Polytechnique de Lausanne (EPFL), Switzerland). LysM^wt/wt^Sirt2^flox/flox^ mice were used as control. The experimental animals were 2–6 months old.

All experiments were performed according to institutional and national guidelines, with local ethical approval in accordance with the recommendations of “Directive 2010/63/EU.”

### Microglia

Primary microglial cell cultures were prepared from individual newborn LysM^Cre/wt^Sirt2^flox/flox^ and control LysM^wt/wt^Sirt2^flox/flox^ mice as described before with a purity >90% (Pais et al., 2008). Briefly, after removal of the meninges, brains were mechanically disrupted in cold Hank’s balanced salt solution (HBSS). Cells were cultured in high-glucose Dulbecco’s Modified Eagle Medium (DMEM) containing Glutamax (Invitrogen) and supplemented with 10% FBS (Endotoxin <10 EU/ml), 5 μg/ml insulin (Sigma), 2.0 mg/ml L-glucose (Sigma), 0.25 ng/ml Granulocyte-macrophage colony-stimulating factor (GM−CSF) (Peprotech) and 1% Penicillin-Streptomycin 50 mM (Invitrogen) for 10 days. Medium was changed after 3 days and at day 6, an equal amount of medium was added to the culture. The confluent mixed glial cell cultures were shaken for 2 h at 250 r.p.m. and microglial cells obtained from the supernatant after centrifugation.

Adult microglia were obtained from brains of LysM^Cre/wt^Sirt2^flox/flox^ and control LysM^wt/wt^Sirt2^flox/flox^ mice (8 weeks old) perfused with 30 ml of PBS via the left ventricle of the heart as described before (Pais and Chatterjee, 2005). Brains were removed and homogenized in HBSS containing collagenase VIII (0.2 mg/ml) (Sigma). After incubation for 45 min at 37°C, the digested tissue was minced, strained trough a nylon cell strainer of 100 μm (BD Falcon^TM^) and centrifuged. The pellet was ressuspended in 30% Percoll (Amersham Biosciences AB, Upssala, Sweden) in PBS and centrifuged at 515 *g* for 30 min at room temperature. Microglial cells were obtained after MACS Cell separation of the pelleted cells incubated with CD11b (Microglia) MicroBeads (Milteny Biotec) according to manufacturer’s instructions. The purity of isolated microglial cells by MACS assessed by CD11b staining and flow cytometry analysis is within 80–90%.

### Western Blot Analysis

Western blot analysis was performed to confirm that microglial expression of Sirt2 was decreased in primary cultures and in adult microglia isolated from LysM^Cre/wt^Sirt2^flox/flox^ in comparison with cells obtained from control LysM^wt/wt^Sirt2^flox/flox^ mice (Supplementary Figure 1). Primary microglial cells and brain adult microglia extracts were prepared in NP-40 lysis buffer (150 mM NaCl, 50 mM Tris–HCl, 1% NP-40 for cell culture; 50 mM Tris, 1 mM EDTA, 5 mM MgCl2, 1% Triton X-100 for brain tissue) containing protease and phosphatase inhibitors (Roche Diagnostics GmbH) and resolved in 12% SDS gels. Proteins were transferred onto nitrocellulose membranes (Bio−Rad) and incubated with primary antibodies against Sirt2 (Sigma) and β-actin (Applied Biosystems) or NF-κB p65 (acetyl K310) (Abcam) followed by incubation with horseradish peroxidase-conjugated secondary antibodies (GE Healthcare). The immunoreactivity was detected with an Immobilon Western Chemiluminescent HRP Substrate (Millipore).

### Hippocampal Slice Preparation

LysM^Cre/wt^Sirt2^flox/flox^ and control LysM^wt/wt^Sirt2^flox/flox^ mice were sacrificed under sevoflurane anaesthesia (Merial, Harlow, United Kingdom) and brains rapidly removed. The skull was exposed by cutting the skin at the top of the head, the cerebrum bisected along the midline, separating the two hemispheres, and both hippocampi dissected in ice-cold, oxygenated (95% O_2_, 5% CO_2_) Kreb’s solution (artificial cerebrospinal fluid, aCSF) containing (in mM): 124 NaCl, 3 KCl, 1.25 NaH_2_PO_4_, 26 NaHCO_3_, 1 MgSO_4_, 2 CaCl_2_, and 10 mM glucose, pH 7,4. Hippocampal slices (400 μm thick) were cut perpendicularly to the long axis of the hippocampus using a McIlwain tissue chopper, and incubated in a submerged resting chamber, with Kreb’s solution, at room temperature, for at least 1 h prior to experimentation, for functional and energetic recovery.

Hippocampal slices, after resting for 1 h at room temperature, were incubated at room temperature, either with LPS (10 μg/ml; Escherichia coli serotype 055:B5, Sigma) or with control BSA (0,002%; Bovine serum albumin) for 20 min.

To assess the role of NMDA receptors activation (namely the extra-synaptic ones), the hippocampal slices were incubated, after resting, first with memantine (1 μM, for 60 min). After this first incubation step, the hippocampal slices from LysM^Cre/wt^Sirt2^flox/flox^ and control LysM^wt/wt^Sirt2^flox/flox^ mice were subsequently exposed either to LPS or control BSA.

### *Ex vivo* Microelectrophysiological Recordings: LTP Induction, Registration and Quantification

Slices were transferred to a recording chamber for submerged slices and continuously superfused at 3 ml/min with Kreb’s solution at 32°C, gassed with 5% CO_2_ and 95% O_2_.

Recordings were obtained with an Axoclamp 2B amplifier and digitized (Axon Instruments, Foster City, CA, United States). Individual responses were monitored, and averages of six consecutive responses were continuously stored on a personal computer with the LTP program (Anderson and Collingridge, 2001). Field excitatory post-synaptic potentials (fEPSPs) were recorded through microelectrode (2–6 MΩ resistance) placed in the stratum radiatum of the CA1 area. Recording electrodes were pulled from borosilicate glass capillary tubes (Harvard Apparatus, United States) and filled with aCSF.

In LTP experiments, stimulation (rectangular 0.1 ms pulses, once every 10 s) was delivered alternatively to two independent pathways through two bipolar concentric electrodes placed on the Shaffer collateral/commissural fibers in the stratum radiatum. The intensity of the stimulus was adjusted to obtain a fEPSP slope within 50% of the maximum slope recorded and having minimal contamination with population spikes.

A stable baseline of at least 20 min was recorded prior to application of theta-burst stimulation (TBS) protocol, which consisted of 10 bursts of 4 stimuli at 100 Hz, separated by 200 ms. The intensity of the stimulus was never changed during these induction protocols. LTP was quantified as the % of change in the average slope of the fEPSP taken from 50 to 60 min after LTP induction in relation to the average slope of the fEPSP measured during the 10 min that preceded the induction of LTP.

Data were acquired using WinLTP 2.01 M,X-Series software and are presented graphically as mean percentage EPSP slope ± standard error of the mean (SEM).

### Input-Output Curve

Input–output curves (I/O) in slices from different genotype groups were performed to ensure that modifications in LTP magnitude were not due to changes in basal synaptic transmission. After obtaining a stable baseline for at least 15 min, the stimulus delivered to the slice (rectangular 0.1 ms pulses, once every 15 s) was decreased until disappearance of the fEPSPs. The stimuli delivered to the slice were then progressively increased by steps of 20 mA. For each stimulation condition, data from three consecutive averaged fEPSP (each averaged fEPSP is the computerized mean of six individual fEPSP) were stored. The range of all inputs delivered to the slice was typically from 60 mA to supra-maximum stimulation amplitude of 300 mA. The input–output curve was plotted as the relationship of fEPSP slope vs stimulus intensity, providing a measure of synaptic efficiency. The max slope (Emax) values were obtained by extrapolation upon nonlinear fitting of the I/O curve.

### Statistical Analysis

Values are reported as mean ± standard deviation (SD). Significant differences between different genotypes and treatments were assessed by means of two-way ANOVA with Bonferroni’s post-hoc correction using IBM SPSS Statistics for Macintosh, Version 25.0. Armonk, NY: IBM Corp. Values of *p* < 0.05 were considered to represent statistically significant differences.

## Results

### LPS Induces LTP Impairment in Hippocampal Slices Deficient in Microglial Sirt2

**FIGURE 1:**
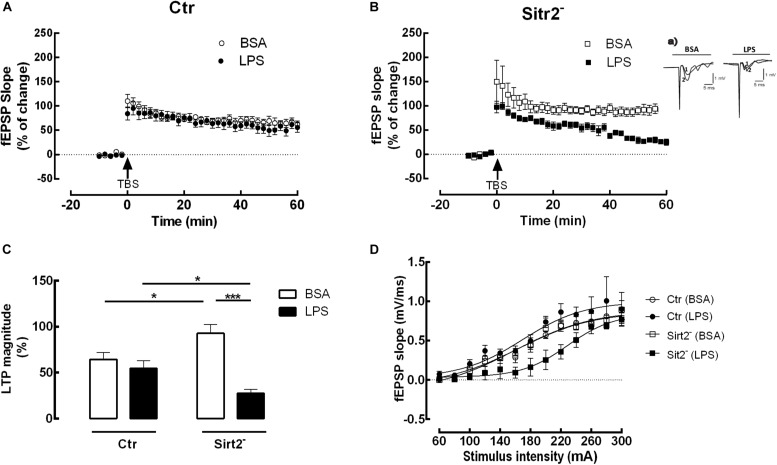
Impaired LTP in microglial Sirt2-deficient hippocampal slices incubated with LPS. Changes in fEPSP slope induced by TBS in hippocampal slices from Ctr **(A)** or Sirt2^–^**(B)** mice pre-treated for 20 min with either vehicle BSA (*n* = 6, ○ and □) or LPS (*n* = 6, 🌑 and ■). Arrow indicates TBS. **(Ba)** shows traces obtained in a representative experiment in **(B)**; each trace is the average of six consecutive responses obtained immediately before (1) and after (2) TBS, and is composed of the stimulus artifact, followed by the presynaptic volley and the fEPSP. **(C)** LTP magnitude % of change, resulting from the difference between the fEPSP slope of LTP induced by TBS during the last 50–60 min of the experiment, in relation to pre-theta-burst values from the experiments shown in **(A)** and **(B)**. Lines indicate statistical significant differences between groups, using two-away ANOVA between-groups with Bonferroni’s post-hoc correction. All values are mean ± SEM, *p < 0.05; ****p < 0.001. **(D)** I/O curves of hippocampal slices stimulated or not with LPS. I/O curves were performed in the presence of either BSA or LPS in hippocampal slices deficient in Sirt2 (BSA, *n* = 4, □; LSP, *n* = 3, ■) and control slices (BSA, *n* = 4, ○; LSP, *n* = 5, 🌑).

Theta-burst-stimulation (TBS) reliably induced a robust and reproducible LTP in both hippocampal slices of mice deficient in microglial Sirt2 (Sirt2^–^) and control mice (Ctr) when incubated either with BSA as a control or with LPS (Figures 1A,B). Two-way analysis of variance revealed a significant main effect for treatment, *F*(1,20) = 23.23, *p* < 0.0001, and a significant interaction between treatment and group, *F*(1,20) = 12.56, *p* < 0.0001. Bonferroni post hoc test was conducted and evidenced a statistically significantly LTP magnitude decrease in slices from Sirt2^–^ mice when incubated with LPS in comparison to BSA (LTP-Sirt2^–^_LPS_ = 27.43 ± 10.26 %, *n* = 6 vs LTP-Sirt2^–^_BSA_ = 92.65 ± 24.07 %, *n* = 6; *p* = 0.001), while there was no significant difference between LPS and BSA-incubated slices of Ctr mice (*p* > 0.05) (Figure 1C). Therefore, lower levels of Sirt2 in microglia strongly reduced the LTP magnitude of hippocampal slices incubated with LPS (Figure 1B). Additionally, LTP magnitude was significantly increased in BSA-treated slices from Sirt2^–^ mice in comparison to slices from Ctr mice (LTP-Sirt2^–^_BSA_ = 92.65 ± 24.07 %, *n* = 6 vs LTP-Ctr_BSA_ = 64.44 ± 18.57 %, *n* = 6; *p* = 0.019) (Figure 1C). Moreover, LTP magnitude recorded in hippocampal slices from Sirt2^–^ mice exposed to LPS was significantly reduced when compared to LTP magnitude recorded in similar conditions in hippocampal slices from Ctr mice (LTP-Sirt2^–^_LPS_ = 27.43 ± 10.26 %, *n* = 6 vs LTP-Ctr_LPS_ = 54.49 ± 20,75 %, *n* = 6; *p* = 0.023) (Figure 1C).

I/O curves confirmed that, using slices taken from the same animals and exposed to LPS in the same conditions of those used in LTP recordings, the alterations detected in LTP magnitude were not attributed to changes in baseline synaptic efficiency (Figure 1D).

Although the I/O curve of hippocampal slices deficient in microglial Sirt2 and exposed to LPS revealed a slight deviation from the other curves, fEPSP values (taken as a measure of the strength of the post-synaptic response) were similar for the four different conditions at the maximum stimulation (Emax): LTP-Ctr_BSA_ = 1.021 ± 0.262, *n* = 4; LTP-Sirt2^–^_BSA_ = 1.028 ± 0.261, *n* = 4; LTP-Ctr_LPS_ = 1.116 ± 0.262, *n* = 5; LTP-Sirt2^–^_LPS_ = 0.909 ± 0.261, *n* = 3. Two-way analysis of variance revealed no significant differences in Emax obtained from slices taken from the different groups. There was no significant main effect of the group, *F*(1,10) = 0.001, *p* = 0.981, neither of treatment, *F*(1,10) = 0.001, *p* = 0.981, nor of the interaction effect between the two *F*(1,10) = 0.838, *p* = 0.381.

Importantly, we attested that the basal stimulation was adjusted to obtain 50% of the maximum fEPSP slope. Therefore, we have used the following intensities for LTP recording: LTP-Ctr_BSA_ = 187 mA; LTP-Sirt2^–^_BSA_ = 184 mA; LTP-Ctr_LPS_ = 182 mA; LTP-Sirt2^–^_LPS_ = 235 mA. Accordingly, the LTP was induced in each hippocampal slice when similar values of fEPSP slope were obtained by adjusting the initial intensity of stimulation. The intensity of the stimulus was maintained during the induction protocol. Therefore, the slices from LTP-Sirt2^–^_LPS_ were stimulated in a range of mA out from the range of stimulation where a decreased response was detected, avoiding therefore any possible influence in LTP induction. Indeed, two-way analysis of variance did not show significant differences between groups regarding the fEPSP slope values corresponding to 50% of the maximum fEPSP slope (*p* > 0.05).

### Memantine Reverts the LTP Decline Induced by LPS in Microglial-Specific Sirt2 Deficient Hippocampal Slices

NMDA receptors are critical for CA1 hippocampal LTP induction and expression (Bliss and Collingridge, 1993; Collingridge et al., 2004). However, excessive or prolonged activation of NMDA receptors can be involved in pathological processes (Rothman and Olney, 1995; Parsons et al., 1998). Accordingly, numerous attempts to reduce glutamate-induced excitotoxicity by antagonizing NMDA receptors have been tested (Sun et al., 2019). However, due to the important physiological role of NMDA receptor activation, most NMDA receptor antagonists produce undesired effects on LTP and memory (Frankiewicz et al., 2000). One of the drugs that has been used to selectively block the pathological activation of NMDA receptors, whilst leaving their physiological functions intact, is memantine (for review see Danysz et al., 1997; Parsons et al., 1999; Danysz and Parsons, 2003; Wenk, 2006). Therefore, in order to investigate the putative role of NMDA receptors in the alterations observed in LTP magnitude in hippocampal slices from microglial-specific Sirt2 deficient mice exposed to LPS, hippocampal slices were pre-incubated with memantine (1 μM, for 60 min) prior to the incubation with LPS or BSA.

We found that pre-incubation with memantine before LPS exposure restored the LTP of LPS-stimulated hippocampal slices to levels similar do the BSA condition (Sirt2^–^_BSA_ = 105.3 ± 18.04%, *n* = 4 vs LTP-Sirt2^–^_Memantine__+__LPS_ = 77.6 ± 16.97%, *n* = 8; *p* > 0.05). In fact, the LTP magnitude of the hippocampal slices from Sirt2^–^ mice incubated with memantine and LPS, was significantly increased when compared to LTP magnitude of hippocampal slices incubated with LPS alone (LTP-Sirt2^–^_Memantine__+__LPS_ = 77.6 ± 16.97%, *n* = 8 vs LTP-Sirt2^–^_LPS_ = 27.8 ± 10.30%, *n* = 6; *p* < 0.001). The pre-incubation of hippocampal slices from Sirt2^–^ mice with memantine followed by BSA incubation did not affect LTP magnitude when compared to BSA incubation alone.

**FIGURE 2:**
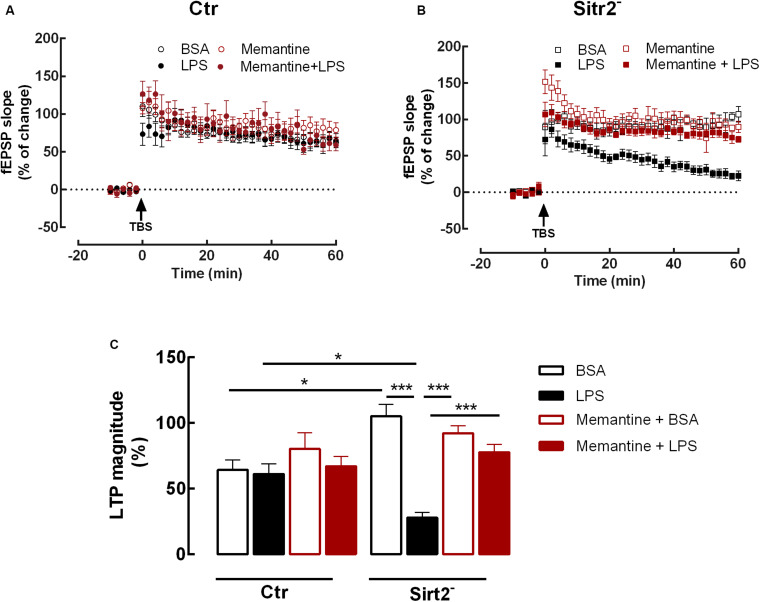
Memantine rescues LTP impairment in microglial Sirt2 deficient hippocampal slices. Changes in fEPSP slope induced by TBS in hippocampal slices from Ctr **(A)** or Sirt2^–^ **(B)** mice pre-treated for 20 min with either vehicle BSA (*n* = 6, ○ and *n* = 4, □) or LPS (*n* = 8, 🌑 and *n* = 6, ■) or for 60 min of memantine followed by 20 min with either vehicle BSA (*n* = 7, 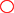 and *n* = 6, 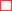) or LPS (*n* = 4, 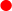 and *n* = 8, 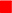). Arrow indicates TBS. **(C)** Plot of LTP magnitude % of change, resulting from the difference between the fEPSP slope of LTP induced by TBS during the last 50–60 min of the experiment, in relation to pre-theta-burst values from the experiments shown in **(A)** and **(B)**, as indicated below the columns. All values are mean ± SEM. Lines indicate statistical significant differences between groups, using two-away ANOVA between-groups with Bonferroni’s post-hoc correction. *p < 0.05; ***p < 0.001.

There were no statistically significant differences regarding LTP magnitude between the four different conditions (BSA, LPS, Memantine+BSA, Memantine+LPS) recorded in hippocampal slices prepared from Ctr mice (*p* > 0.05) (Figure 2).

## Discussion

In the present study, we show a significant impairment on LTP magnitude in hippocampal slices from microglia-specific Sirt2 deficient mice pre-exposed to the general inflammatory stimulus LPS.

Sirt2 has already been shown to hold a key-inhibitory role in microglia-mediated inflammation (Pais et al., 2013). In the current study, we propose that Sirt2 may also take part in protective mechanisms that ensure CNS synaptic transmission and plasticity under an inflammatory environment.

Microglia processes are driven by neuronal activity and can simultaneously interact with both presynaptic and postsynaptic elements (Wake et al., 2009). In response to inflammatory stimuli, microglia are activated, releasing key regulatory molecules, such as TNF-α, IL-1β, IL-6, and IL-8, prostaglandin E2, as well as glutamate, reactive oxygen and nitrogen species, that are known to be highly upregulated during CNS inflammation and to control synaptic transmission and plasticity (Milner and Campbell, 2003; Di Filippo et al., 2008; Dilger and Johnson, 2008; Gomes-Leal, 2012). However, whether and how microglia influence physiological synaptic transmission is still unclear. Nevertheless, there is evidence that under pathological conditions, activation of microglia, which is the primary stage of neuroinflammation, is a common early feature of most brain diseases, followed by synaptic alterations (Cagnin, 2001; Dekosky and Marek, 2003).

According to our published work, microglia not expressing Sirt2, upon an inflammatory stimulus like LPS, become over-activated due to the absence of NF-κB inhibition by Sirt2 (Pais et al., 2013). This leads to overproduction of pro-inflammatory cytokines, such as TNF-α, IL-6, and reactive oxygen and nitrogen species (Pais et al., 2013), all known to have a key role in synaptic plasticity and to mediate LTP impairment (Wang et al., 2015; Rizzo et al., 2018).

To induce deletion of the floxed Sirt2 in microglia, we have used the Cre under the LysM promoter that has been shown to induce a limited recombination in microglia (∼40%) when compared to the CX3CR1 promoter (∼90%) (Goldmann and Prinz, 2013). Although we observed a partial decrease in microglial Sirt2 levels (Supplementary Figure 1) it would be interesting to perform the LTP experiments in hippocampal slices of CX3CR1^Cre/wt^Sirt2^flox/flox^ mice which will probably have a stronger *Sirt2* gene deletion.

We found an increased LTP magnitude in hippocampal slices from microglial Sirt2 deficient mice under control conditions. A possible explanation might be that, in microglia-specific Sirt2 deficient mice, in absence of Sirt2, hippocampal microglia become “primed,” being able to easily liberate and produce important factors for synaptic plasticity. Although these might be beneficial at a certain level, when overproduced, as it may happen after LPS stimulation they may contribute to LTP decrease. This seems the case of IL-1β, which has a dual role in LTP, being required for LTP under physiological conditions, but inhibiting LPT when at higher doses, as encountered in pathological conditions (Ross et al., 2003). Our finding contrasts, however, with the results of a recent study that has shown that whole-body Sirt2 knockout (KO) mice present an LTP reduction in hippocampal slices without any inflammatory stimulation, which is accompanied by impairments in long-term memory and is likely result from enhanced acetylation and surface expression of AMPA receptors in neurons (Wang et al., 2017). Future work will investigate whether our observation is due to selective deficiency of Sirt2 in microglia.

Here we show that the absence of Sirt2 specifically in microglia is responsible for an LTP impairment under inflammatory conditions. Interestingly, the I/O curve of hippocampal slices deficient in microglial Sirt2 and exposed to LPS revealed a slight deviation from the others, which may suggest a certain latency in achieving maximal synaptic response. However, there were no significant differences regarding fEPSP slope values between groups, neither for the initial intensity of stimulation before LTP induction, neither for the obtained maximal synaptic responses between slices (Emax). This finding is supported by a previous study that has also proved that loss of Sirt2 did not cause changes in basal synaptic properties compared to Ctr mice (Wang et al., 2017).

Rescue of LTP impairment with memantine in LPS stimulated microglial Sirt2-deficient hippocampal slices strongly suggests the involvement of extra-synaptic NMDA receptors. Although CA1 LTP depends upon synaptic NMDA receptors activation (Fox et al., 2006), extra-synaptic NMDA receptors activation are well-known for mediating toxic effects (Viviani et al., 2006; Xia et al., 2010). Extra-synaptic NMDARs are enriched in GluN2B-containing heterodimers (GluN2B-NMDARs) and its activity has been associated with neuronal death, in particular in neurodegenerative disorders such as Huntington’s or Alzheimer’s disease (Papouin and Oliet, 2014). In fact, memantine is an antagonist of the NMDA receptors, and at the concentration used in this study particularly inhibits extra-synaptic NMDA receptors (Xia et al., 2010). These extra-synaptic NMDA receptors are generally activated by pro-inflammatory and neurotoxic factors produced by activated microglia, such as IL-1β, TNF and glutamate (Barger and Basile, 2001; Viviani et al., 2003; Floden et al., 2005; Gardoni et al., 2011). Both IL-1β and TNF were shown to decrease LTP (Cunningham et al., 1996; Coogan and OConnor, 1997; Murray and Lynch, 1998; Ross et al., 2003). Several studies have reported a cross-talk between activation of IL-1β and NMDA receptors (Viviani et al., 2003; Yang et al., 2005; Gardoni et al., 2011). In fact, IL-1RI (interleukin-1 receptor type I) colocalizes with the GluN2B subunit of NMDA extra-synaptic receptors in hippocampal neurons (Gardoni et al., 2011). Importantly, pre-treatment of hippocampal neurons with IL-1β enhance NMDA-induced [Ca^2 +^]i through activation of the Src family of kinases, which increase tyrosine phosphorylation of the NR2A/B subunits (Viviani et al., 2003). IL-1β may have a similar effect during LTP induction as it also involves Src kinase activation (Salter, 1998). Although it is clear that IL-1β and other inflammatory factors interfere with NMDA receptors, the effect in calcium influxes may be dose dependent and have different outcomes in physiological and pathological conditions. We have previously shown that Sirt2-deficent microglia become overactivated by LPS stimulation. Therefore, we hypothesize that LPS-incubated hippocampal slices of microglial-specific Sirt2 deficient mice overproduce inflammatory mediators such as IL-1β, TNF and glutamate enhancing NMDA-induced neuronal death. Future investigation of the specific mechanisms underlying the decreased LTP will be needed to identify the precise factors released by Sirt2-deficient microglia in stimulated hippocampal slices. Moreover, we cannot exclude an anti-inflammatory effect of memantine. Besides being an antagonist of the NMDA receptors, memantine has also been shown to inhibit microglial activation by LPS (Rosi et al., 2006; Wu et al., 2009).

Given the crucial role of microglia in neuroinflammation, the fact that Sirt2 is able to regulate the inflammatory response in the CNS through microglial cells, with an impact in synaptic plasticity, makes it a potential therapeutic target to prevent cognitive decline associated to neuroinflammation. Although development of new drugs for the prevention of neurodegenerative diseases has been an area of intense activity, little progress has been observed. Interestingly, various potent small-molecule modulators of sirtuins have shown efficacy in preclinical models of neurodegenerative and inflammatory diseases, holding promise for drug discovery efforts in multiple therapeutic areas (Lavu et al., 2008; Langley and Sauve, 2013; Min et al., 2013). Our data reinforces this possibility and, in particular, point toward microglial Sirt2 as a potential therapeutic target to prevent cognitive decline associated to neuroinflammation.

## Conclusion

The results obtained show that, in the absence of microglial Sirt2, inflammatory stimuli significantly impair NMDA-mediated synaptic plasticity. This reveals a key-protective role for microglial Sirt2 in neuroinflammation.

By showing neuroprotective effects, Sirt2 emerges as a potential target for therapeutic intervention in an array of neurological disorders, playing a vital role in all conditions where microglial activation and neuroinflammation may have a major importance in the disease process, like neurodegenerative diseases. This matter is of great importance, since the comprehension of the molecular mechanisms underlying sirtuins activity may help us find new therapeutic targets for these diseases.

## Data Availability Statement

The raw data supporting the conclusions of this article will be made available by the authors, without undue reservation, to any qualified researcher upon request.

## Ethics Statement

The animal study was reviewed and approved by by the iMM’s Institutional Animal Welfare Body – ORBEA-iMM and the National Competent Authority – DGAV (Direção-Geral de Alimentação e Veterinária).

## Author Contributions

TP, MD, and AS contributed conception and design of the study. JS and MV performed the main experiments. JF-G, ST, RB, and CM-L contributed to the experimental work. JS, TP, and MD wrote the manuscript. All authors read and approved the submitted version.

## Conflict of Interest

The authors declare that the research was conducted in the absence of any commercial or financial relationships that could be construed as a potential conflict of interest.
