# Supplementary material for: Microglial Sirtuin 2 Shapes Long-Term Potentiation in Hippocampal Slices
Source: Front Neurosci. 2020 Jun 18;14:614. doi: 10.3389/fnins.2020.00614 (PMC7315392; doi:10.3389/fnins.2020.00614)
Supplement: Supplementary file 1 [file Data_Sheet_1.docx]

**Supplementary Material**

**Supplementary Figures**


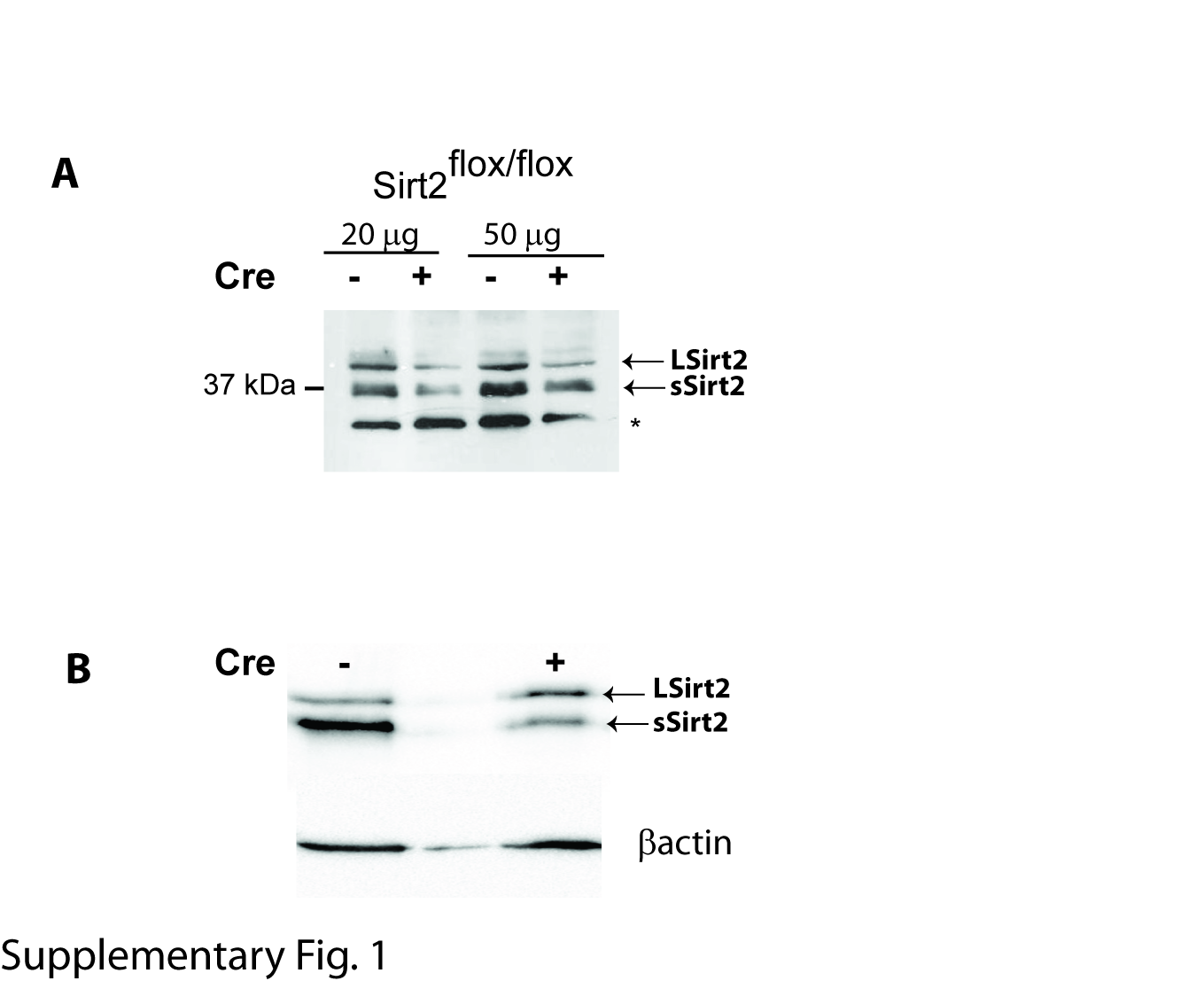


Figure S1: Levels of Sirt2 in primary and adult microglial cells. A) Western blot analysis of primary cultures and B) adult microglia prepared from brains of LysM^Cre/wt^Sirt2^flox/flox^ (Cre^+^) and LysM^wt/wt^Sirt2^flox/flox^ (Cre^-^) control mice. LSirt2 and sSirt2 indicate the large and short isoforms of Sirt2, respectively. * Indicates a non-specific band
